# Epithelioid schwannoma of the skin displaying unique histopathological features: a teaching case giving rise to diagnostic difficulties on a morphological examination of a resected specimen, with a brief literature review

**DOI:** 10.1186/s13000-017-0604-9

**Published:** 2017-01-19

**Authors:** Sohsuke Yamada, Mari Kirishima, Tsubasa Hiraki, Michiyo Higashi, Kazuhito Hatanaka, Akihide Tanimoto

**Affiliations:** 1 Department of Pathology, Field of Oncology, Graduate School of Medical and Dental Sciences, Kagoshima University, 8-35-1 Sakuragaoka, Kagoshima, 890-8544 Japan; 2 Department of Pathology, Kagoshima University Hospital, Kagoshima, 890-8544 Japan

**Keywords:** Epithelioid schwannoma, Rare variant, Skin, S-100 protein

## Abstract

**Background:**

Epithelioid schwannoma as a rare variant poses a challenge to all pathologists, as this uncommon entity is extremely difficult to conclusively diagnose by morphological analyses on a resected sample alone owing to its unique histopathological features. However, few papers have described the detailed clinicopathological characteristics of epithelioid schwannoma.

**Case presentation:**

A 65-year-old female presented with a history of a flat and slightly elevated firm and tan plaque accompanied by occasional tenderness, measuring 10 × 8 mm, in the right joint of her hand 1 year before resection. A gross examination of a locally resected specimen revealed an encapsulated nodular lesion, yellow-whitish in color, partly filled with blood. A microscopic examination showed that the tumor predominantly consisted of a solid proliferation of epithelioid cells having mildly enlarged and round to partially spindled nuclei and abundant vacuolated or clear cytoplasm with very few mitotic figures and modest nuclear size variation, associated with focal hyalinized, cystic and hemorrhagic degeneration. This well-demarcated tumor was surrounded by dense, hyalinized and layered fibrocollagenous stroma. Immunohistochemically, these tumor cells were diffusely positive for S-100 protein and had a very low MIB-1 labeling index, and type IV collagen was strongly reactive with reduplicated basal lamina of them. We ultimately made a diagnosis of cutaneous epithelioid schwannoma.

**Conclusion:**

We should be aware that, since pathologists might misinterpret epithelioid schwannoma as other soft tissue tumors, including its malignant counterpart, a wide panel of immunohistochemical antibodies can be powerful supplementary tools for identifying a very rare entity of conventional schwannoma.

## Background

Among cutaneous soft tissue neoplasms, benign nerve sheath tumor, i.e. schwannoma, is a very common and well-known entity; however, epithelioid schwannoma is a very rare and relatively new morphologic variant of benign schwannoma, composed predominantly of histopathologically characteristic epithelioid schwann cells [1–4]. Epithelioid schwannoma was first described by Orosz et al. and Kindblom et al. in the early 1990s [3, 4], and subsequently, Hart et al. recently reviewed their experience with up to 58 cases of it in more detail [2]. Our thorough investigation showed that, to date, merely less than 80 cases of true epithelioid schwannoma have been reported in the English literature, as summarized in Tables 1 to 2.

**Table 1:** Summary of clinical data and histomorphologic

Authors	N	Age (yr)	Sex (N)	Location (N)	Size (cm)	Symptoms	Followup/Interval (N)	Component of conventional schwannoma (N)	Mitotic activity (N)	Cytologicatypia (N)
Orosz et al. [3]	1	42	F	Back	1.5	Slowly growing subcutaneous nodule	NED/15 months (1)	None	“Inconspicuous”	None
Kindbolm et al. [4]	5	23–73	M (4), F (1)	Exteremity (3), face (1), neck of urinary bladder (1)	1–4.5	Painless palpable mass (4), urinary obstructive symptoms and hematuria (1)	NED/2 months to 2 years (5)	Transition to spindled areas of classic schwannoma (1)	Zero to one mitosis/10 HPF	Focally “cells with larger and more hyperchrom atic nuclei suggesting symplastic change”
Hart et al. [2]	58	14–80	M (31), F (26)	Extremity (40), thorax/abdomen/back (10), scalp (3), neck (2), lip (1), breast (1)	0.25–4.5	NG	NED/NG (39), Recurrence/N G (1), Lost/NG (1)	“Epithelioid areas focally and gradually modulating with spindled areas and palisaded nuclei” (20); “other features of classic schwannomas with hyalinized and ectatic vessels” (55)	≧3 mitoses/10 HPF (20)	“Significant nuclear atypia” (13)
Yamada et al.	1	65	F	Extremity	1	Subcutaneo us nodule with tenderness	NED/9 months (1)	None	≦1 mitosis/50 HPF	None

*N*, case number, *NED* no evidence of disease, *NG* not given or available, *HPF* high-power field

**Table 2:** Summary of immunohistochemical profiles of the neoplastic cells in epithelioid schwannoma (positivity)

Authors	S-100 protein	Vimentin	CKs	EMA	α-SMA	Desmin	CD10	CD31	CD34	CD68	p63	Type IV collagen	Melan-A	HMB-45	CDK4	MDM2	p53	GFAP	Ki67 (MIB-1)
Orosz et al. [3]	1/1	1/1	0/1	0/1	0/1	0/1	NG	NG	NG	NG	NG	NG	NG	NG	NG	NG	NG	0/1	NG
Kindbol m et al. [4]	5/5	5/5	0/5	3/5 (s)	0/5	NG	NG	NG	0/2	NG	NG	NG	NG	0/5	NG	NG	0/5	1/5 (F)	≦1% (4/5), 1-10% (1/5,F)
Hart et al. [2]	56/56	NG	2/48 (F)	NG	NG	NG	NG	NG	NG	NG	NG	16/17	16/17 1/24 (W)	NG	NG	NG	NG	4/6	NG
Yamada et al.	1/1	1/1	0/1	0/1	0/1	0/1	0/1	0/1	0/1	0/1	0/1	0/1	0/1	0/1	0/1	0/1	0/1	NP	≦1%

*CK* cytokeratins, *NG* not given or available, *F* focally, *W* weakly, *s* subcapsular, *NP* not performed

Clinically, epithelioid schwannoma of the skin may simulate various benign to malignant neoplasms of the skin, including pyogenic granuloma and basal cell carcinoma or squamous cell carcinoma, because all of these lesions can look tan and reddish on the surface [1–4]. In the largest series investigating this entity to date (Table 1), the most common anatomic location of epithelioid schwannoma was the extremities, with involvement of the forearm, hand and finger/thumb [2]. Epithelioid schwannoma is defined conceptually as the benign counterpart of epithelioid malignant peripheral nerve sheath tumor (MPNST) [1–4] and frequently poses a diagnostic challenge to all clinicians and pathologists, as this very rare entity is extremely difficult to diagnose by a morphological examination alone, i.e. haematoxylin and eosin (H&E) staining, on a resected sample due to its unique histopathological features, in marked contrast to conventional schwannoma [1–4]. Indeed, its histological differential diagnoses widely include true epithelial neoplasms and myoepithelial, epithelioid mesenchymal and neuroectodermal lesions [1, 2], as is summarized in Table 3. Further hampering an accurate diagnosis, somewhat atypical histological features might result in a misdiagnosis as epithelioid MPNST, which shows a much worse prognosis than benign epithelioid schwannoma [2]. The early accurate diagnosis and conservative treatment of epithelioid schwannoma might allow patients an improved quality of life. However, very few reports have described its detailed clinicopathological features.

**Table 3:** Immunohistological features of epithelioid schwannoma and the other tumors of its differential diagnoses

	S-100 protein	Vimentin	CKs	EMA	α-SMA	Desmin	CD10	CD31	CD34	CD68	p63	Type IV collagen	Melan-A	HMB-45	CDK4	MDM2	p53	Ki67 (MIB-1)
Epithelioid schwannoma	+	+	-	-	-	-	-	-	-	-	-	+	-	-	-	-	-	Very low
Epithelioid MPNST	+	+	-	-	-	-	-	-	-	-	-	+	-	-	-	-	+	High
Soft tissue myoepithelioma	+	+	+	+	F+	F+	F+	-	-	-	F+	+	-	-	-	-	-	Very low
Epithelioid hemangioendothelioma	-	+	F+	-	-	-	F+	+	+	-	-	+	-	-	-	-	NG	Low to intermediate
Perineurioma of sclerosing or reticular variants	-	+	-	+	-	-	+	-	-	-	-	+	-	-	-	-	-	Very low
Epithelioid leiomyoma	-	+	-	-	+	+	+	-	-	-	+	-	-	-	-	-	-	Very low
Malignant melanoma	+	+	-	-	-	-	+	-	-	-	-	-	+	+	-	-	+	Very high
Liposarcoma, dedifferentiated	-	+	-	-	-	-	-	-	-	-	-	-	-	-	+	+	+	HIgh

*MPNST* malignant peripheral nerve sheath tumor, *CK* cytokeratins, *F* focally, *NG* not given or available

We herein report an extremely rare case of benign epithelioid schwannoma displaying unique histopathological features originating from the superficial subcutaneous soft tissue of the joint of the right hand, together with presentation of a review of the literature. The microscopic examination via H&E staining did not allow for a conclusive diagnosis, and the diagnosis was ultimately made based on a wide panel of immunohistochemical analyses, including one of its hallmark antibodies, S-100 protein.

## Case presentation

**Fig. 1:**
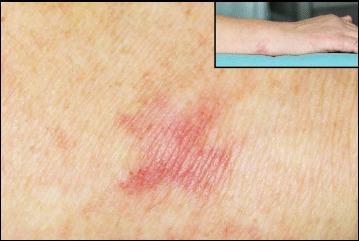
The clinical findings at surgery of the epithelioid schwannoma specimen. A 65-year-old female presented with a history of a very slow-growing, flat and slightly elevated firm and tan plaque accompanied by occasional tenderness, measuring approximately 10 × 8 mm, in the ulnar joint of the right hand (inset), covered by mostly smooth epidermis

A 65-year-old female presented with a history of a very slow-growing, flat and slightly elevated firm and tan plaque covered by mostly a smooth epidermis and accompanied by occasional tenderness measuring approximately 10 × 8 mm in the ulnar joint of the right hand, 1 year before resection (Fig. 1). She had an unremarkable medical history. The laboratory data, including the blood cell count, chemistry, and tumor markers, were unavailable. There were no imaging analyses, either. The dermatologists first interpreted this lesion as a benign tumor, such as a pilomatricoma, but could not completely rule out malignancy.

**Fig. 2:**
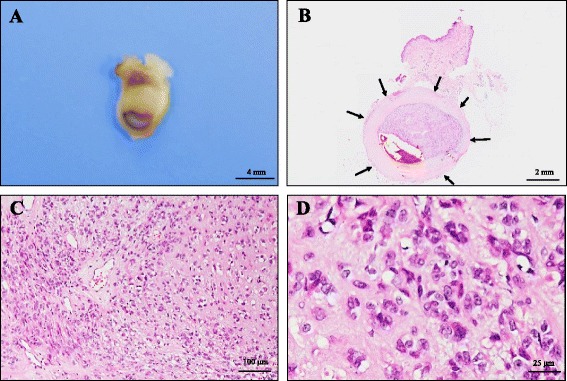
The gross and microscopic findings of the resected specimen of epithelioid schwannoma arising from the superficial subcutaneous soft tissue. **a** Tumor extirpation was performed, and a gross examination of the cut surface revealed an encapsulated, well-demarcated nodular lesion, yellow-whitish in color, partly filled with blood, measuring 5 × 4 mm in diameter. Bar = 4 mm. **b** On scanning magnification (H&E stain), this superficial subcutaneous tumor was found to contain a peripheral cyst-like, degenerative cavity filled with red blood cells and showed an encapsulated and well-circumscribed nodule surrounded by dense, hyalinized and layered fibrocollagenous stroma (arrows), reminiscent of a vascular-like structure. Bar = 2 mm. **c** Under a low-power view, this tumor predominantly comprised a solid proliferation of characteristic epithelioid cells, embedded partially in a small amount of hyalinized and/or myxoid stroma. Scattered small blood vessels intervened in this epithelioid schwannoma. Neither an infiltrative appearance nor necrotic foci were evident. Bar = 100 μm (H&E stain) (original magnification: × 100). **d** Under a high-power view, these neoplastic cells revealed mildly atypical epithelioid cells with mildly enlarged, pleomorphic and round to partially spindled, polygonal nuclei and abundant vacuolated or clear cytoplasm with very few mitotic figures (much less than 1/50 high-power fields), less than three nuclear size variations, and frequent intranuclear pseudo-inclusions. Bar = 25 μm (H&E stain) (original magnification: × 400)

Local resection for tumor extirpation was performed, and a gross examination of its cut surface displayed an encapsulated, well-demarcated nodular lesion, yellow-whitish in color, partly filled with blood, measuring 5 × 4 mm in diameter (Fig. 2a). On scanning magnification, this subcutaneous tumor was found to contain a peripheral cyst-like cavity filled with red blood cells and showed an encapsulated and well-circumscribed nodule, uniquely surrounded by dense, hyalinized and layered fibrocollagenous stroma, reminiscent of vascular-like structure (Fig. 2b). Resection was diagnosed as complete by this histopathological examination.

**Fig. 3:**
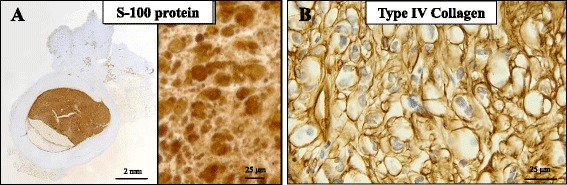
The immunohistochemical examination of the resected specimen of cutaneous epithelioid schwannoma. **a** On scanning magnification (lt.) of immunohistochemistry (S-100 protein), this encapsulated vessel wall-like stroma contained no apparent schwann cells. Furthremore, immunohistochemical staining of S-100 protein could not detect any elements of apparent contiguous native perineurium of the peripheral nerve to this well-demarcated epithelioid schwannoma (Bar = 2 mm). The epithelioid tumor cells were diffusely positive for S-100 protein (rt.; Bar = 25 μm [original magnification: × 400]). **b** Type IV collagen was strongly reactive with reduplicated basal lamina originating from the differentiated epithelioid schwann cells (original magnification: × 400)

Microscopically, as summarized in Table 1, the tumor predominantly comprised a solid proliferation of characteristic epithelioid cells (Fig. 2c) having mildly enlarged, pleomorphic and round to partially spindled, polygonal nuclei and abundant vacuolated or clear cytoplasm with very few mitotic figures (much less than 1/50 high-power fields) (Fig. 2d), embedded partially in a small amount of hyalinized and/or myxoid stroma and associated with focal cystic and hemorrhagic degeneration (Fig. 2b, c). Scattered small blood vessels intervened in this tumor (Fig. 2c). Neither an infiltrative appearance nor necrotic foci were evident. Under a high-power view, these neoplastic cells revealed mildly atypical epithelioid cells, with less than three nuclear size variations, and frequent intranuclear pseudo-inclusions (Fig. 2d). Elastica van Gieson (EVG) staining could not detect any elastic fibers in this encapsulated vessel-like structure. No apparent conventional schwannoma features, including Antoni A and/or B areas or rosette formations, were noted. Furthermore, there were no elements of apparent contiguous native perineurium of the peripheral nerve to this well-demarcated tumor, within our thorough investigation, as confirmed by an immunohistochemical analysis using S-100 protein (diluted 1:2,000; Dako Cytomation Co., Glostrup, Denmark) (Fig. 3a).

Immunohistochemically, the tumor cells were diffusely positive for vimentin (diluted 1:1; Dako) and S-100 protein (Dako) (Fig. 3a), and type IV collagen (diluted 1:200; Dako) was strongly reactive with reduplicated basal lamina of them (Fig. 3b). By contrast, they were completely negative for cytokeratins (CKs) (AE1/AE3, diluted 1:1; Dako; & Cam5.2, diluted 1:10; Becton Dickinson Immunocytometry Systems, San Jose, CA, USA), EMA (diluted 1:1; Dako), α-smooth muscle actin (α-SMA, diluted 1:1; Dako), desmin (diluted 1:1; Dako), CD10 (diluted 1:1; Dako), CD31 (diluted 1:1; Dako), CD34 (diluted 1:1; Dako), CD68 (KP-1, diluted 1:200; Dako), p63 (diluted 1:200; Dako), Melan A (diluted 1:50; Dako), HMB45 (diluted 1:100; Dako), CDK4 (diluted 1:50; Santa Cruz Biotechnology, Dallas, TX, USA), MDM2 (diluted 1:200; Invitrogen, Carlsbad, CA, USA) and p53 (diluted 1:1,000; Dako). The immunohistochemical profiles of these epithelioid cells in the present case are summarized in Table 2. In addition, the Ki67 (MIB-1, diluted 1:1; Dako) labeling index was much less than 1% in the proliferating tumor cells. Finally, immunohistochemical staining of S-100 protein could not detect any reactive elements, including contiguous native perineurium of the peripheral nerve, in this encapsulated vessel-like structure (Fig. 3a). All of the immunohistochemical stainings were conducted using the Dako Envision kit (Dako) in accordance with the manufacturer’s instructions [5–8].

Based on these features, we ultimately made a diagnosis of epithelioid schwannoma arising from the superficial subcutaneous soft tissue of the ulnar joint of the right hand. To date, after approximately 9 months of post-operative follow-up, the patient remains well with neither recurrence nor metastases.

## Discussion

As presented in Tables 1 to 2, the few case reports and review articles available on epithelioid schwannomas have suggested that a confident and accurate diagnosis distinguishing this entity from other benign soft tissue tumors and epithelioid MPNST might be impossible based on a morphological (H&E staining) examination alone, since not only do these lesions have diverse histopathological features, but a lack of experience and/or misinterpretation of findings by physicians can prove to be a stumbling block [1–4].

Therefore, we agree with previous studies indicating the importance of applying a wide panel of immunohistochemical antibodies in the conclusive diagnosis of round to spindle cell neoplasms of the skin [2, 5]. Among benign tumors, the histological differential diagnoses of the current case included soft tissue myoepithelioma, epithelioid hemangioendothelioma, perineurioma of sclerosing or reticular variants, or epithelioid leiomyoma, as summarized in Table 3. Immunohistochemical analyses generally resolve these distinctions easily. As shown in Table 3, based on the immunopositivity of the S-100 protein and the negative expressions of p63, actins (α-SMA), epithelial markers (AE1/AE3, Cam5.2, and EMA), CD31, CD34, and desmin, we were able to exclude the possibilities of the above neoplasms. In addition, as presented in Fig. 3b, it is well known that type IV collagen is a confirmative surrogate marker of reduplicated basal lamina originating from differentiated schwann cells [2]. In addition to distinguishing the lesion from more recently proposed atypical variants of epithelioid schwannoma [1, 2], further analyses of the low MIB-1 labeling indices and the negative expression of p53 on the resected specimen would be very powerful supplementary tools for excluding the possibility of malignancy (epithelioid MPNST) (Table 3).

It is very likely that the present case report of epithelioid schwannoma arising in the superficial subcutaneous tissue in the right forearm is histopathologically remarkable for one reason at least: the present tumor was surrounded by dense, hyalinized and layered fibrocollagenous stroma, reminiscent of vascular-like structures. Although conventional schwannoma usually exhibits hyalinized and thick-walled small- to medium-sized vessels in its stroma [1, 2], this characteristic finding in our case is very uncommon. In fact, there were no overt features of classic, conventional schwannoma, including Antoni A and/or B areas or rosette formations, and this encapsulated vessel wall-like stroma contained neither EVG-positive elastic fibers nor S-100 protein-positive schwann cells. Furthermore, as shown in Fig. 3a, immunohistochemical staining of S-100 protein could not detect any elements of apparent contiguous native perineurium of the peripheral nerve to this well-demarcated epithelioid schwannoma. This unusual feature might have resulted from severely degenerative changes of the present epithelioid schwannoma. However, further investigation will be required to resolve this intriguing issue after the accumulation of its similar surgical cases.

## Conclusions

We herein reported a very rare case of epithelioid schwannoma arising from the superficial subcutaneous soft tissue of the ulnar joint of the right hand that proved very difficult to tentatively diagnose based solely on a morphological examination of a resected specimen. However, we were subsequently able to accurately diagnose the lesion after thorough analyses of the immunohistochemistry. All pathologists should be aware that the characteristic features of this lesion might result in a diagnostic pitfall. An appropriate and wide panel of immunohistochemical antibodies, including various schwann cell, myoepithelial, endothelial, perineurial, and smooth muscle cell markers, p53 and MIB-1 labeling index, can therefore be powerful supplementary tools for identifying benign lesions and reaching the correct, final diagnosis of cutaneous epithelioid schwannoma.

## Abbreviations

MPNST: Malignant peripheral nerve sheath tumor
